# Editorial: Recent advances in the development of vaccines against *Acinetobacter baumannii*

**DOI:** 10.3389/fimmu.2023.1187554

**Published:** 2023-05-31

**Authors:** Saeed Khalili, Wangxue Chen, Abolfazl Jahangiri

**Affiliations:** ^1^ Department of Biology Sciences, Shahid Rajaee Teacher Training University, Tehran, Iran; ^2^ Human Health Therapeutics Research Center (HHT), National Research Council Canada, Ottawa, ON, Canada; ^3^ Department of Biology, Brock University, St. Catharines, ON, Canada; ^4^ Applied Microbiology Research Center, Systems Biology and Poisonings Institute, Baqiyatallah University of Medical Sciences, Tehran, Iran

**Keywords:** vaccine, active immunization, antigen, protection, immunogen, *Acinetobacter baumannii*

*Acinetobacter baumannii* is a notorious Gram-negative healthcare-associated (nosocomial and community-acquired) bacterial pathogen that causes a high mortality rate (up to 70%). The rapid emergence of highly antibiotic-resistant strains is a further propellant to introduce the bacterium as a serious public health threat. Hence, the World Health Organisation (WHO) listed *A. baumannii* (carbapenem-resistant) as the first priority that needs new antibiotics. The recent coronavirus disease 2019 (COVID-19) pandemic further increased the outbreak of carbapenem-resistant *A. baumannii* infections. Many novel antibiotics were introduced into the market but few are effective against *A. baumannii*. Hence, further approaches such as immunization trials had been considered as alternative solutions against *A. baumannii* infections. Several antigens had been introduced for active and passive immunizations. An effective robust immunization should develop full protection against various types of infections and all pathogenic strains. Moreover, it should have no deleterious effect on microbiota as well as the human proteome. Despite rigorous basic and pre-clinical studies conducted on active and passive immunizations against this pathogen, no vaccine has been advanced to clinical trials. Recently, the combination of protective antigens (1, 2), epitopes/peptides (3) or presentation of protective epitopes by appropriate scaffold (4) had been suggested as effective promising approaches against *A. baumannii*.

In this Research Topic, 4 research articles and one review article on recent advances in the development of vaccines against *A. baumannii* are collected.

Sun et al. conducted a study in which a 39-amino acid extracellular peptide from C-terminal of Ata was fused to the cholera toxin B subunit (CTB), as immunoadjuvant. The construct elicited both Th1 and Th2 immune responses in mice with no overt inflammation. Immunized mice showed a significant decrease in the blood and tissue bacterial loads. A broad protection against a variety of *A. baumannii* strains was also reported. Moreover, the addition of CpG to the aluminum adjuvant further enhanced the immune response, especially cellular immunity. The findings introduced the developed antigen as a promising vaccine candidate against *A. baumannii* infection.

The research article by Chaudhuri et al. evaluated if novel hybrid constructs presenting external loops of Baumannii acinetobactin utilization A (BauA) could confer protection against *A. baumannii* infection. BauA is the outer membrane receptor for the siderophore acinetobactin, and commercial production of membrane proteins for vaccine development is challenging. Hence, in this study, three immunogenic exposed loops (5, 7 and 8) of BauA were selected and incorporated individually or in combination into loopless C-lobe (LCL), a scaffold derived from the C-lobe of *Neisseria meningitidis* transferrin binding protein B (TbpB). The designed constructs were evaluated in an *A. baumannii* murine sepsis model. The mice immunized with the recombinant BauA protein or the designed hybrid antigen loop 7 showed complete protection against infection whereas the other hybrid antigens conferred <100% protection. Since the LCL scaffold did not confer protection, the observed protection could be attributed to the displayed BauA loops. These results suggest that the loop 7 of BauA could be an appropriate immunogenic peptide to combat *A. baumannii* infections.

Another research article of this Research Topic collection is about the identification of immunodominant vaccine candidates in *A. baumannii* by a subtractive proteomics approach [Acar et al.]. This study was conducted based on the hypothesis that IgGs from infected patients could recognize immunodominant epitopes/proteins. IgGs obtained from healthy (as control) and patient sera were used to capture the secreted and outer membrane proteins of drug-resistant and sensitive *A. baumannii*. The captured proteins were analyzed by Liquid Chromatography-Tandem Mass Spectrometry (LC-MS/MS). Patient sera captured 34 unique proteins of drug-resistant *A. baumannii*.

The study reported by Dollery et al. in this Research Topic assessed the efficacy of radiation-inactivated whole-cell vaccine candidates against *A. baumannii* infection. Utilization of the ultraviolet C (UVC)-irradiation, instead of gamma-irradiation, for vaccine inactivation was the most salient feature of this study. UVC is considered an easier and more accessible inactivation method for vaccine inactivation. Moreover, they performed a high dose and two boost vaccination regimen to assess the effects of manganous-decapeptide-phosphate (MDP) complex for the protection of antigen epitopes from oxidative damage. However, the obtained results revealed that UVC-inactivated vaccines act equivalently in the presence and absence of MDP during irradiation. Results from the passive vaccination with immune sera indicate that a humoral response is sufficient for the protection of neutropenic mice in this. In light of these observations, UVC-based vaccine inactivation could be an amenable approach to prepare whole-cell vaccine candidates against *A. baumannii.*

The review by Tan and Lahiri focuses on contemporary efforts in unveiling the promising *A. baumannii* vaccine candidates and drug targets using recent approaches. Aside from the conventional trial-and-error approaches, this study explains the pro and cons of incorporating in silico methods in recent vaccine development studies against *A. baumannii*. They discussed the role of in silico analyses, particularly network analysis, in recognition of novel vaccine candidates in the proteome of *A. baumannii*. They showed that in silico approaches, such as building co-functional networks and their analyses by k-shell decomposition, network-based centrality measurements, molecular docking and molecular dynamics simulation on structural data, have already been harnessed for the successful unveiling of novel *A. baumannii* vaccine candidates and drug targets. Given these circumstances, it seems that in silico approaches would have an ever-growing role in the design and discovery of vaccines and drugs against *A. baumannii* infections.

In conclusion, the five reports published in this Research Topic collection provide an excellent update and summary of the current status of the preclinical development of *A. baumannii* vaccines. As Antimicrobial Resistance (AMR) is now at the forefront of the global public health agenda and *A. baumannii* is the 5^th^ leading bacterial pathogen responsible for deaths associated with AMR in 2019 (https://www.thelancet.com/journals/lancet/article/PIIS0140-6736(21)02724-0/fulltext), we hope this Research Topic will stimulate new and additional research into the host immune responses and antigen discovery with an ultimate goal to develop safe and efficient vaccines against *A. baumannii* infections. Whereas several logistic issues in the clinical development of vaccines for some AMR pathogens remain, with the recent advances and success in the development of COVID-19 vaccines, together with the increased awareness of the AMR crisis and the support from different levels of governments and industries we are reasonably optimistic that the availability of *Acinetobacter* vaccines as an additional arsenal for clinical management of *A. baumannii* infection will become reality soon.

## Author contributions

AJ contributed to conception and design of the study. SK and AJ wrote the first draft of the manuscript. WC revised and finalized the manuscript. All authors contributed to manuscript revision, read, and approved the submitted version.
